# [5,10,15,20-Tetra­kis(4-chloro­phen­yl)porphyrinato]bis­(tributyl­phosphine)cobalt(III) perchlorate

**DOI:** 10.1107/S1600536809019163

**Published:** 2009-05-29

**Authors:** Bijan Etemadi, Reza Kia, Mozaffar Asadi, Kh. Mohammadi

**Affiliations:** a Department of Earth Sciences, Faculty of Sciences, Shiraz University, Shiraz 71454, Iran; b Chemistry Department, Faculty of Sciences, Shiraz University, Shiraz 71454, Iran; c Chemistry Department, Faculty of Sciences, Persian Gulf University, Bushehr 75169, Iran

## Abstract

In the mol­ecule of the title compound, [Co(C_44_H_24_Cl_4_N_4_){(C_4_H_9_)_3_P}_2_]ClO_4_, the Co^III^ centre has a slightly distorted octa­hedral geometry and is coordinated by four N atoms of the tetra­pyrrolic ring in the equatorial positions and two phosphine ligands in the axial positions. The dihedral angles between *meso*-substituted chloro­phenyl rings and the basic tetra­pyrrolic ring are 82.66 (9), 82.16 (7), 83.97 (11) and 76.87 (8)°. In one of the phosphine ligands, the two terminal methyl groups are disordered over two positions with refined site-occupancy ratios of 0.70 (7):0.30 (7) and 0.66 (2):0.34 (2). In the crystal structure, mol­ecules are linked together along the *a* axis by inter­molecular C—H⋯Cl inter­actions. The crystal structure is further stabilized by intra­molecular C—H⋯O and C—H⋯N inter­actions and inter­molecular C—H⋯O and C—H⋯π inter­actions.

## Related literature

For bond-length data, see Allen *et al.* (1987 ▶). For applications of metalloporphyrins in organic synthesis, see: Liu *et al.* (2007 ▶). For the synthesis, see: Mohammadi (2005 ▶).
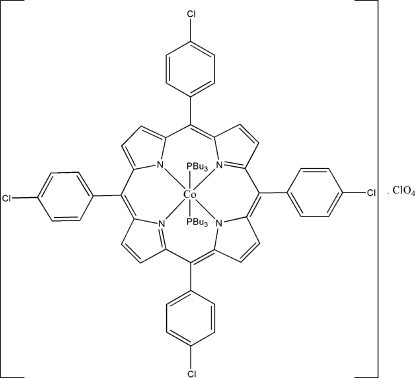


## Experimental

### 

#### Crystal data

[Co(C_44_H_24_Cl_4_N_4_)(C_12_H_27_P)_2_]ClO_4_
*M* *_r_* = 1313.46
Monoclinic, 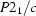

*a* = 12.4803 (5) Å
*b* = 21.8192 (8) Å
*c* = 24.0902 (10) Å
β = 99.542 (3)°
*V* = 6469.2 (4) Å^3^
*Z* = 4
Mo *K*α radiation
μ = 0.57 mm^−1^
*T* = 120 K
0.50 × 0.20 × 0.14 mm

#### Data collection

Stoe IPDS-II diffractometer
Absorption correction: numerical shape of crystal determined optically (*X-RED32*; Stoe & Cie, 2005 ▶)*T*_min_ = 0.869, *T*_max_ = 0.918
41515 measured reflections
17260 independent reflections
13604 reflections with *I* > 2σ(*I*)
*R*_int_ = 0.091

#### Refinement

*R*[*F* ^2^ > 2σ(*F* ^2^)] = 0.069
*wR*(*F* ^2^) = 0.181
*S* = 1.08
17260 reflections
773 parameters
4 restraints
H-atom parameters constrained
Δρ_max_ = 0.74 e Å^−3^
Δρ_min_ = −0.76 e Å^−3^

### 

Data collection: *X-AREA* (Stoe & Cie, 2005 ▶); cell refinement: *X-AREA*; data reduction: *X-AREA*; program(s) used to solve structure: *SHELXTL* (Sheldrick, 2008 ▶); program(s) used to refine structure: *SHELXTL*; molecular graphics: *SHELXTL*; software used to prepare material for publication: *SHELXTL* and *PLATON* (Spek, 2009 ▶).

## Supplementary Material

Crystal structure: contains datablocks global, I. DOI: 10.1107/S1600536809019163/at2788sup1.cif

Structure factors: contains datablocks I. DOI: 10.1107/S1600536809019163/at2788Isup2.hkl

Additional supplementary materials:  crystallographic information; 3D view; checkCIF report

## Figures and Tables

**Table 1:** Selected geometric parameters (Å, °)

Co1—N4	1.979 (2)
Co1—N2	1.985 (2)
Co1—N1	1.989 (2)
Co1—N3	1.997 (2)
Co1—P2	2.3385 (9)
Co1—P1	2.3395 (8)

**Table 2:** Hydrogen-bond geometry (Å, °)

*D*—H⋯*A*	*D*—H	H⋯*A*	*D*⋯*A*	*D*—H⋯*A*
C22—H22⋯O3^i^	0.95	2.48	3.403 (5)	165
C31—H31⋯O3	0.95	2.55	3.277 (5)	134
C32—H32⋯O1	0.95	2.49	3.407 (5)	163
C34—H34⋯Cl4^ii^	0.95	2.80	3.643 (4)	148
C50—H50*B*⋯N4	0.99	2.59	3.359 (4)	134
C67—H67*B*⋯O1	0.99	2.52	3.376 (6)	145
C66—H66*B*⋯*Cg*1	0.99	2.43	3.335 (4)	151
C53—H53*A*⋯*Cg*2	0.99	2.89	3.574 (4)	127
C58—H58*A*⋯*Cg*3	0.99	2.48	3.431 (4)	161
C50—H50*B*⋯*Cg*4	0.99	2.47	3.353 (4)	149
C61—H61*B*⋯*Cg*4	0.99	2.77	3.428 (3)	125

Symmetry codes: (i) 
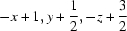
; (ii) 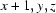. *Cg*1, *Cg*2, *Cg*3, and *Cg*4 are the centroids of the N1/C2–C5, N2/C7–C10, N3/C12–C15, and N4/C17–C20 rings, respectively.

**Fig. 1.**
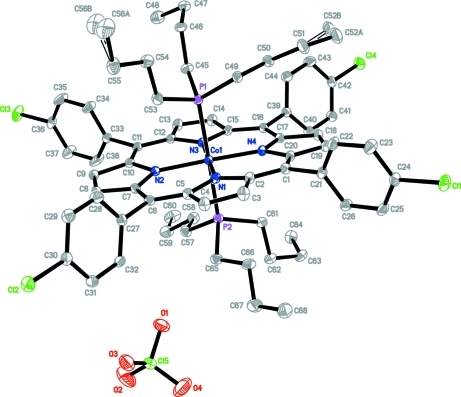
The molecular structure of the title compound, showing 30% probability displacement ellipsoids and the atomic numbering. Hydrogen atoms were omitted for clarity. Open bonds show the minor component.

**Fig. 2.**
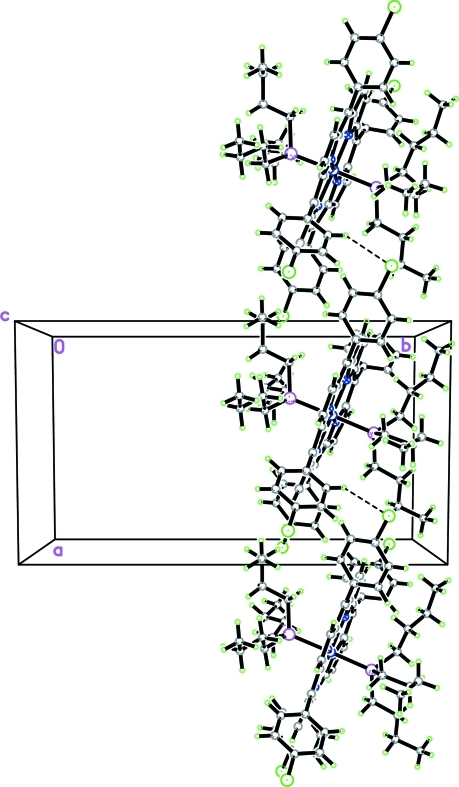
The crystal packing of the major component of the title compound, viewed down the c-axis, showing linking of the molecules along the a-axis through intermolecular C—H···Cl interactions. Intermolecular interactions are drawn as dashed lines.

## supplementary crystallographic information

### Comment

Metalloporphyrins exist ubiquitously in nature and have found a broad spectrum of applications. Among the different applications, great efforts have been made to develop new complexes as catalysts in organic transformation reactions (Liu, *et al.* 2007).

The molecule of the title compound, Fig. 1, is a substituted metallo- porphyrin. In the title compound, the Co atom has a slightly distorted octahedral geometry which is coordinated by four nitrogen atoms in the equatorial position of the tetrapyrrolic ring and two other phosphine ligands in the axial positions with P1–Co1–P2, P1–Co1–N1 and N1–Co1–N3 angles of 179.18 (13), 88.48 (7), and 178.85 (10)°, respectively (Table 1). The dihedral angles between *meso*- substituted chlorophenyl rings and the basic tetrapyrrol ring are 82.66 (9), 82.16 (7), 83.97 (11), and 76.87 (8)°. In one of the phosphine ligands the two terminal methyl groups are disordered over two positions with a refined site-occupancies ratio of 0.70 (7)/0.3 (7) and 0.66 (2)/0.34 (2). In the crystal structure, the molecules are linked together by the intermolecular C—H···Cl interactions along the *a* axis (Fig. 2). The crystal structure is further stabilized by the intramolecular C—H···O and C—H···N and the intermolecular C—H···O and C—H···π interactions [*Cg*1, *Cg*2, *Cg*3, and *Cg*4 are the centroids of the N1/C2–C5, N2/C7–C10, N3/C12–C15, and N4/C17–C20 five-membered pyrrole rings] (Table 2).

### Experimental

In a round-bottomed flask the porphyrinatocobalt(II) (1 mol) was dissolved in methanol (100 ml) and then PBu_3_ (2 mol) was added to the solution. After heating, the reaction mixture was oxidized by blowing air into the solution for 1 h and the reaction mixture was checked for completing by UV-vis spectrophotometry. After completing, sodium perchlorate (1.2 mol) was added and by evaporating solvent, the resulting brown product was collected. Single crystals suitable for *X*-ray diffraction were obtained from methanol solution.

### Refinement

All of the hydrogen atoms were positioned geometrically [C—H = 0.95–0.99 Å] and refined using a riding model approximation with U_iso_ (H) = 1.2 or 1.5 U_eq_ (C). A rotating group model was applied to the methyl groups. In one of the phosphine ligands the two terminal methyl groups are disordered over two positions with a refined site-occupancies ratio of 0.70 (7)/0.3 (7) and 0.66 (2)/0.34 (2).

The bond distances of the major and minor components were restrained to be 1.54 (1) Å.

### Crystal data

**Table:**

[Co(C_44_H_24_Cl_4_N_4_)(C_12_H_27_P)_2_]ClO_4_	*F*(000) = 2752
*M**_r_* = 1313.46	*D*_x_ = 1.349 Mg m^−^^3^
Monoclinic, *P*2_1_/*c*	Mo *K*α radiation, λ = 0.71073 Å
Hall symbol: -P 2ybc	Cell parameters from 1298 reflections
*a* = 12.4803 (5) Å	θ = 3.1–29.2°
*b* = 21.8192 (8) Å	µ = 0.57 mm^−^^1^
*c* = 24.0902 (10) Å	*T* = 120 K
β = 99.542 (3)°	Block, brown
*V* = 6469.2 (4) Å^3^	0.50 × 0.20 × 0.14 mm
*Z* = 4	

### Data collection

**Table:**

Stoe IPDS-II diffractometer	17260 independent reflections
Radiation source: fine-focus sealed tube	13604 reflections with *I* > 2σ(*I*)
graphite	*R*_int_ = 0.091
Detector resolution: 0.15 pixels mm^-1^	θ_max_ = 29.3°, θ_min_ = 1.7°
rotation method scans	*h* = −17→16
Absorption correction: numerical shape of crystal determined optically	*k* = 0→29
*T*_min_ = 0.869, *T*_max_ = 0.918	*l* = 0→32
17260 measured reflections	

### Refinement

**Table:**

Refinement on *F*^2^	Primary atom site location: structure-invariant direct methods
Least-squares matrix: full	Secondary atom site location: difference Fourier map
*R*[*F*^2^ > 2σ(*F*^2^)] = 0.069	Hydrogen site location: inferred from neighbouring sites
*wR*(*F*^2^) = 0.181	H-atom parameters constrained
*S* = 1.08	*w* = 1/[σ^2^(*F*_o_^2^) + (0.0701*P*)^2^ + 9.0268*P*] where *P* = (*F*_o_^2^ + 2*F*_c_^2^)/3
17260 reflections	(Δ/σ)_max_ = 0.002
773 parameters	Δρ_max_ = 0.74 e Å^−^^3^
4 restraints	Δρ_min_ = −0.76 e Å^−^^3^

### Special details

**Table:**

Experimental. (program? reference?)
Geometry. All esds (except the esd in the dihedral angle between two l.s. planes) are estimated using the full covariance matrix. The cell esds are taken into account individually in the estimation of esds in distances, angles and torsion angles; correlations between esds in cell parameters are only used when they are defined by crystal symmetry. An approximate (isotropic) treatment of cell esds is used for estimating esds involving l.s. planes.
Refinement. Refinement of F^2^ against ALL reflections. The weighted R-factor wR and goodness of fit S are based on F^2^, conventional R-factors R are based on F, with F set to zero for negative F^2^. The threshold expression of F^2^ > 2sigma(F^2^) is used only for calculating R-factors(gt) etc. and is not relevant to the choice of reflections for refinement. R-factors based on F^2^ are statistically about twice as large as those based on F, and R- factors based on ALL data will be even larger.

### Fractional atomic coordinates and isotropic or equivalent isotropic displacement parameters (Å^2^)

**Table:**

	*x*	*y*	*z*	*U*_iso_*/*U*_eq_	Occ. (<1)
Co1	0.38297 (3)	0.731732 (17)	0.918800 (14)	0.01758 (9)	
Cl1	−0.04116 (9)	0.89313 (5)	0.59051 (4)	0.0456 (2)	
Cl2	0.95841 (7)	0.62065 (5)	0.72825 (4)	0.0405 (2)	
Cl3	0.83243 (8)	0.62163 (5)	1.25879 (4)	0.0479 (2)	
Cl4	−0.21035 (7)	0.85554 (4)	1.08795 (4)	0.03853 (19)	
P1	0.45653 (6)	0.82911 (3)	0.94063 (3)	0.02090 (14)	
P2	0.31178 (6)	0.63377 (3)	0.89761 (3)	0.02301 (15)	
N1	0.4121 (2)	0.74303 (11)	0.84075 (9)	0.0209 (4)	
N2	0.52919 (19)	0.69466 (11)	0.94034 (9)	0.0201 (4)	
N3	0.35557 (19)	0.72185 (11)	0.99768 (9)	0.0196 (4)	
N4	0.23561 (19)	0.76654 (11)	0.89676 (9)	0.0195 (4)	
C1	0.2392 (2)	0.79044 (13)	0.79671 (11)	0.0226 (5)	
C2	0.3451 (2)	0.77074 (14)	0.79673 (11)	0.0231 (5)	
C3	0.3958 (3)	0.77163 (16)	0.74726 (12)	0.0294 (6)	
H3	0.3667	0.7890	0.7118	0.035*	
C4	0.4931 (3)	0.74300 (16)	0.76082 (12)	0.0288 (6)	
H4	0.5448	0.7360	0.7367	0.035*	
C5	0.5027 (2)	0.72517 (14)	0.81919 (11)	0.0235 (5)	
C6	0.5919 (2)	0.69390 (13)	0.84806 (11)	0.0220 (5)	
C7	0.6031 (2)	0.68083 (13)	0.90556 (11)	0.0219 (5)	
C8	0.6994 (2)	0.65403 (14)	0.93724 (11)	0.0240 (5)	
H8	0.7610	0.6396	0.9227	0.029*	
C9	0.6855 (2)	0.65337 (14)	0.99187 (11)	0.0238 (5)	
H9	0.7361	0.6391	1.0230	0.029*	
C10	0.5798 (2)	0.67833 (13)	0.99367 (11)	0.0211 (5)	
C11	0.5342 (2)	0.68264 (13)	1.04294 (11)	0.0205 (5)	
C12	0.4285 (2)	0.70203 (13)	1.04315 (10)	0.0210 (5)	
C13	0.3801 (3)	0.70369 (16)	1.09331 (12)	0.0282 (6)	
H13	0.4138	0.6920	1.1301	0.034*	
C14	0.2774 (3)	0.72507 (15)	1.07818 (12)	0.0276 (6)	
H14	0.2251	0.7309	1.1022	0.033*	
C15	0.2627 (2)	0.73723 (13)	1.01848 (11)	0.0220 (5)	
C16	0.1684 (2)	0.76223 (13)	0.98752 (11)	0.0218 (5)	
C17	0.1572 (2)	0.77464 (13)	0.93003 (11)	0.0217 (5)	
C18	0.0608 (2)	0.80118 (14)	0.89788 (12)	0.0254 (6)	
H18	−0.0043	0.8113	0.9114	0.031*	
C19	0.0805 (2)	0.80901 (14)	0.84491 (12)	0.0252 (6)	
H19	0.0318	0.8256	0.8141	0.030*	
C20	0.1890 (2)	0.78753 (13)	0.84395 (11)	0.0222 (5)	
C21	0.1737 (2)	0.81530 (14)	0.74356 (11)	0.0239 (5)	
C22	0.1873 (3)	0.87534 (16)	0.72700 (14)	0.0340 (7)	
H22	0.2418	0.9004	0.7480	0.041*	
C23	0.1211 (3)	0.89881 (17)	0.67964 (14)	0.0383 (8)	
H23	0.1297	0.9400	0.6685	0.046*	
C24	0.0437 (3)	0.86243 (16)	0.64923 (12)	0.0309 (6)	
C25	0.0300 (3)	0.80183 (16)	0.66358 (13)	0.0330 (7)	
H25	−0.0229	0.7767	0.6415	0.040*	
C26	0.0961 (3)	0.77879 (15)	0.71129 (12)	0.0295 (6)	
H26	0.0879	0.7373	0.7219	0.035*	
C27	0.6818 (2)	0.67604 (14)	0.81741 (11)	0.0236 (5)	
C28	0.7724 (3)	0.71387 (16)	0.81846 (14)	0.0321 (7)	
H28	0.7760	0.7515	0.8385	0.038*	
C29	0.8572 (3)	0.69741 (17)	0.79075 (14)	0.0333 (7)	
H29	0.9187	0.7233	0.7917	0.040*	
C30	0.8499 (3)	0.64217 (16)	0.76158 (12)	0.0280 (6)	
C31	0.7617 (3)	0.60412 (16)	0.75925 (12)	0.0290 (6)	
H31	0.7584	0.5667	0.7389	0.035*	
C32	0.6768 (2)	0.62110 (15)	0.78725 (12)	0.0262 (6)	
H32	0.6153	0.5951	0.7858	0.031*	
C33	0.6057 (2)	0.66694 (14)	1.09760 (11)	0.0221 (5)	
C34	0.6741 (3)	0.71086 (17)	1.12511 (15)	0.0411 (9)	
H34	0.6733	0.7510	1.1098	0.049*	
C35	0.7442 (4)	0.69763 (19)	1.17488 (16)	0.0474 (10)	
H35	0.7913	0.7283	1.1933	0.057*	
C36	0.7443 (3)	0.63962 (16)	1.19687 (12)	0.0294 (6)	
C37	0.6747 (4)	0.59564 (18)	1.17145 (16)	0.0482 (11)	
H37	0.6738	0.5560	1.1876	0.058*	
C38	0.6054 (4)	0.60970 (17)	1.12173 (16)	0.0495 (11)	
H38	0.5569	0.5793	1.1041	0.059*	
C39	0.0766 (2)	0.78129 (14)	1.01628 (11)	0.0229 (5)	
C40	−0.0219 (3)	0.74974 (15)	1.00936 (14)	0.0300 (6)	
H40	−0.0289	0.7122	0.9891	0.036*	
C41	−0.1099 (3)	0.77219 (15)	1.03159 (14)	0.0309 (6)	
H41	−0.1768	0.7505	1.0263	0.037*	
C42	−0.0991 (3)	0.82624 (15)	1.06139 (13)	0.0284 (6)	
C43	−0.0030 (3)	0.85783 (18)	1.07029 (16)	0.0388 (8)	
H43	0.0037	0.8946	1.0917	0.047*	
C44	0.0850 (3)	0.83526 (17)	1.04756 (15)	0.0350 (7)	
H44	0.1517	0.8571	1.0535	0.042*	
C45	0.4623 (3)	0.84880 (14)	1.01521 (12)	0.0288 (6)	
H45A	0.3881	0.8440	1.0241	0.035*	
H45B	0.5087	0.8179	1.0377	0.035*	
C46	0.5035 (3)	0.91218 (14)	1.03605 (13)	0.0297 (6)	
H46A	0.4737	0.9435	1.0079	0.036*	
H46B	0.5837	0.9132	1.0400	0.036*	
C47	0.4697 (3)	0.92759 (17)	1.09295 (15)	0.0365 (7)	
H47A	0.4951	0.9695	1.1041	0.044*	
H47B	0.3894	0.9276	1.0883	0.044*	
C48	0.5142 (4)	0.8835 (2)	1.14015 (14)	0.0439 (9)	
H48A	0.5934	0.8811	1.1435	0.066*	
H48B	0.4826	0.8428	1.1317	0.066*	
H48C	0.4952	0.8984	1.1757	0.066*	
C49	0.3864 (3)	0.89276 (14)	0.90078 (13)	0.0272 (6)	
H49A	0.4382	0.9272	0.9017	0.033*	
H49B	0.3664	0.8797	0.8610	0.033*	
C50	0.2849 (3)	0.91642 (15)	0.92055 (15)	0.0316 (7)	
H50A	0.3052	0.9349	0.9584	0.038*	
H50B	0.2355	0.8816	0.9239	0.038*	
C51	0.2249 (3)	0.96417 (19)	0.88043 (18)	0.0437 (9)	
H51A	0.2712	1.0011	0.8813	0.052*	0.70 (7)
H51B	0.2147	0.9475	0.8417	0.052*	0.70 (7)
H51C	0.1923	0.9457	0.8455	0.052*	0.30 (7)
H51D	0.2734	0.9961	0.8728	0.052*	0.30 (7)
C52B	0.130 (3)	0.986 (3)	0.908 (2)	0.058 (4)	0.30 (7)
H52A	0.0751	1.0060	0.8802	0.087*	0.30 (7)
H52B	0.1568	1.0160	0.9381	0.087*	0.30 (7)
H52C	0.0975	0.9514	0.9250	0.087*	0.30 (7)
C52A	0.1146 (13)	0.9831 (10)	0.8938 (13)	0.058 (4)	0.70 (7)
H52D	0.0656	0.9477	0.8893	0.087*	0.70 (7)
H52E	0.0841	1.0157	0.8680	0.087*	0.70 (7)
H52F	0.1232	0.9980	0.9327	0.087*	0.70 (7)
C53	0.5958 (3)	0.83288 (15)	0.92532 (16)	0.0324 (7)	
H53A	0.6404	0.8039	0.9511	0.039*	
H53B	0.5940	0.8171	0.8866	0.039*	
C54	0.6553 (3)	0.89337 (17)	0.92935 (16)	0.0353 (7)	
H54A	0.6093	0.9242	0.9066	0.042*	
H54B	0.6672	0.9074	0.9690	0.042*	
C55	0.7632 (3)	0.88982 (19)	0.90940 (19)	0.0447 (9)	
H55A	0.7529	0.8686	0.8726	0.054*	0.66 (2)
H55B	0.8133	0.8646	0.9364	0.054*	0.66 (2)
H55C	0.7519	0.8858	0.8692	0.054*	0.34 (2)
H55D	0.8025	0.8545	0.9255	0.054*	0.34 (2)
C56A	0.8163 (8)	0.9517 (4)	0.9031 (6)	0.061 (2)	0.66 (2)
H56A	0.7713	0.9754	0.8735	0.092*	0.66 (2)
H56B	0.8885	0.9454	0.8931	0.092*	0.66 (2)
H56C	0.8234	0.9741	0.9388	0.092*	0.66 (2)
C56B	0.8325 (18)	0.9471 (8)	0.9283 (10)	0.061 (2)	0.34 (2)
H56D	0.7847	0.9825	0.9295	0.092*	0.34 (2)
H56E	0.8822	0.9550	0.9017	0.092*	0.34 (2)
H56F	0.8745	0.9400	0.9659	0.092*	0.34 (2)
C57	0.3621 (3)	0.57427 (15)	0.95002 (13)	0.0296 (6)	
H57A	0.3564	0.5341	0.9306	0.036*	
H57B	0.4402	0.5821	0.9635	0.036*	
C58	0.3052 (3)	0.56885 (16)	1.00171 (13)	0.0328 (7)	
H58A	0.3136	0.6078	1.0231	0.039*	
H58B	0.2266	0.5616	0.9892	0.039*	
C59	0.3534 (4)	0.51626 (18)	1.03979 (15)	0.0449 (9)	
H59A	0.3389	0.4769	1.0195	0.054*	
H59B	0.4332	0.5215	1.0493	0.054*	
C60	0.3047 (4)	0.5144 (2)	1.09388 (16)	0.0535 (12)	
H60A	0.2255	0.5107	1.0844	0.080*	
H60B	0.3232	0.5523	1.1152	0.080*	
H60C	0.3341	0.4792	1.1167	0.080*	
C61	0.1631 (2)	0.63131 (14)	0.89282 (12)	0.0255 (6)	
H61A	0.1452	0.6434	0.9298	0.031*	
H61B	0.1311	0.6625	0.8651	0.031*	
C62	0.1079 (3)	0.56971 (16)	0.87610 (14)	0.0321 (7)	
H62A	0.1490	0.5365	0.8981	0.039*	
H62B	0.1089	0.5619	0.8357	0.039*	
C63	−0.0109 (3)	0.56917 (18)	0.88682 (14)	0.0363 (7)	
H63A	−0.0486	0.6062	0.8696	0.044*	
H63B	−0.0485	0.5329	0.8682	0.044*	
C64	−0.0188 (3)	0.56764 (19)	0.94888 (15)	0.0396 (8)	
H64A	0.0151	0.6045	0.9672	0.059*	
H64B	0.0187	0.5312	0.9662	0.059*	
H64C	−0.0954	0.5662	0.9533	0.059*	
C65	0.3362 (3)	0.59678 (15)	0.83241 (13)	0.0302 (6)	
H65A	0.4153	0.5977	0.8318	0.036*	
H65B	0.3146	0.5532	0.8337	0.036*	
C66	0.2784 (3)	0.62395 (16)	0.77757 (13)	0.0347 (7)	
H66A	0.2062	0.6391	0.7831	0.042*	
H66B	0.3206	0.6595	0.7675	0.042*	
C67	0.2639 (4)	0.5789 (2)	0.72945 (16)	0.0545 (12)	
H67A	0.2123	0.5465	0.7367	0.065*	
H67B	0.3345	0.5591	0.7274	0.065*	
C68	0.2215 (4)	0.6094 (2)	0.67360 (15)	0.0514 (11)	
H68A	0.2755	0.6386	0.6645	0.077*	
H68B	0.1537	0.6311	0.6762	0.077*	
H68C	0.2078	0.5782	0.6440	0.077*	
Cl5	0.52807 (8)	0.45849 (4)	0.74529 (4)	0.0444 (2)	
O1	0.4951 (3)	0.50384 (14)	0.78197 (15)	0.0561 (8)	
O2	0.5615 (4)	0.40497 (17)	0.7797 (2)	0.0828 (13)	
O3	0.6197 (3)	0.48022 (15)	0.72168 (15)	0.0553 (8)	
O4	0.4411 (3)	0.4444 (3)	0.70195 (19)	0.0988 (17)	

### Atomic displacement parameters (Å^2^)

**Table:**

	*U*^11^	*U*^22^	*U*^33^	*U*^12^	*U*^13^	*U*^23^
Co1	0.01903 (18)	0.01944 (17)	0.01378 (15)	0.00064 (13)	0.00131 (12)	0.00126 (12)
Cl1	0.0540 (6)	0.0447 (5)	0.0299 (4)	0.0044 (4)	−0.0171 (4)	0.0088 (3)
Cl2	0.0303 (4)	0.0599 (6)	0.0341 (4)	0.0018 (4)	0.0138 (3)	−0.0098 (4)
Cl3	0.0408 (5)	0.0682 (6)	0.0279 (4)	−0.0050 (4)	−0.0144 (3)	0.0130 (4)
Cl4	0.0267 (4)	0.0418 (5)	0.0500 (5)	0.0055 (3)	0.0149 (3)	−0.0044 (4)
P1	0.0204 (3)	0.0207 (3)	0.0209 (3)	−0.0004 (3)	0.0014 (2)	0.0017 (2)
P2	0.0273 (4)	0.0219 (3)	0.0192 (3)	−0.0002 (3)	0.0019 (3)	−0.0002 (2)
N1	0.0217 (11)	0.0252 (11)	0.0154 (9)	0.0000 (9)	0.0022 (8)	0.0007 (8)
N2	0.0217 (11)	0.0230 (11)	0.0153 (9)	0.0017 (9)	0.0020 (8)	−0.0012 (8)
N3	0.0218 (11)	0.0228 (11)	0.0137 (9)	0.0017 (9)	0.0011 (8)	0.0003 (8)
N4	0.0190 (10)	0.0216 (11)	0.0174 (9)	0.0003 (9)	0.0018 (8)	0.0026 (8)
C1	0.0256 (14)	0.0241 (13)	0.0180 (11)	−0.0006 (11)	0.0027 (10)	0.0049 (10)
C2	0.0255 (14)	0.0275 (14)	0.0159 (11)	−0.0001 (11)	0.0021 (10)	0.0052 (10)
C3	0.0306 (15)	0.0409 (17)	0.0175 (12)	0.0026 (13)	0.0059 (11)	0.0075 (11)
C4	0.0290 (15)	0.0388 (17)	0.0197 (12)	0.0040 (13)	0.0078 (11)	0.0041 (11)
C5	0.0253 (14)	0.0267 (13)	0.0192 (11)	−0.0010 (11)	0.0050 (10)	0.0014 (10)
C6	0.0247 (13)	0.0229 (13)	0.0185 (11)	0.0019 (11)	0.0042 (10)	−0.0015 (9)
C7	0.0229 (13)	0.0215 (13)	0.0212 (12)	0.0016 (10)	0.0035 (10)	0.0003 (10)
C8	0.0226 (13)	0.0280 (14)	0.0213 (12)	0.0052 (11)	0.0037 (10)	−0.0005 (10)
C9	0.0251 (14)	0.0266 (14)	0.0185 (11)	0.0021 (11)	0.0006 (10)	−0.0009 (10)
C10	0.0233 (13)	0.0215 (12)	0.0172 (11)	−0.0005 (10)	0.0001 (10)	0.0007 (9)
C11	0.0243 (13)	0.0210 (12)	0.0155 (11)	−0.0002 (10)	0.0015 (9)	0.0000 (9)
C12	0.0240 (13)	0.0238 (13)	0.0143 (10)	−0.0008 (10)	0.0004 (9)	0.0003 (9)
C13	0.0280 (15)	0.0401 (17)	0.0169 (11)	0.0005 (13)	0.0046 (10)	0.0026 (11)
C14	0.0280 (15)	0.0379 (16)	0.0177 (12)	0.0002 (13)	0.0060 (10)	0.0023 (11)
C15	0.0228 (13)	0.0258 (13)	0.0177 (11)	−0.0013 (11)	0.0043 (10)	−0.0001 (10)
C16	0.0218 (13)	0.0238 (13)	0.0199 (11)	−0.0003 (10)	0.0039 (10)	0.0001 (10)
C17	0.0208 (13)	0.0231 (13)	0.0214 (12)	0.0005 (10)	0.0039 (10)	−0.0010 (10)
C18	0.0224 (13)	0.0284 (14)	0.0251 (13)	0.0022 (11)	0.0028 (10)	0.0011 (11)
C19	0.0233 (13)	0.0281 (14)	0.0225 (12)	0.0021 (11)	−0.0010 (10)	0.0023 (10)
C20	0.0223 (13)	0.0235 (13)	0.0199 (12)	−0.0010 (11)	0.0010 (10)	0.0027 (10)
C21	0.0232 (13)	0.0287 (14)	0.0189 (11)	0.0000 (11)	0.0012 (10)	0.0030 (10)
C22	0.0323 (17)	0.0329 (16)	0.0313 (15)	−0.0062 (13)	−0.0108 (13)	0.0087 (13)
C23	0.044 (2)	0.0342 (17)	0.0319 (16)	−0.0073 (15)	−0.0074 (14)	0.0127 (13)
C24	0.0325 (16)	0.0359 (16)	0.0216 (13)	0.0035 (13)	−0.0033 (11)	0.0039 (12)
C25	0.0365 (17)	0.0349 (17)	0.0234 (13)	−0.0029 (14)	−0.0077 (12)	−0.0007 (12)
C26	0.0367 (17)	0.0284 (15)	0.0212 (12)	−0.0015 (13)	−0.0017 (12)	0.0019 (11)
C27	0.0233 (13)	0.0293 (14)	0.0180 (11)	0.0034 (11)	0.0025 (10)	0.0012 (10)
C28	0.0335 (17)	0.0303 (15)	0.0341 (15)	−0.0051 (13)	0.0105 (13)	−0.0052 (12)
C29	0.0297 (16)	0.0378 (17)	0.0341 (15)	−0.0060 (14)	0.0104 (13)	−0.0022 (13)
C30	0.0248 (14)	0.0400 (17)	0.0196 (12)	0.0036 (13)	0.0047 (10)	0.0002 (11)
C31	0.0311 (16)	0.0340 (16)	0.0218 (13)	0.0021 (13)	0.0038 (11)	−0.0054 (11)
C32	0.0230 (13)	0.0317 (15)	0.0236 (12)	−0.0025 (12)	0.0026 (10)	−0.0040 (11)
C33	0.0229 (13)	0.0258 (13)	0.0173 (11)	−0.0001 (11)	0.0022 (10)	0.0012 (10)
C34	0.050 (2)	0.0313 (17)	0.0338 (16)	−0.0108 (16)	−0.0170 (15)	0.0067 (13)
C35	0.057 (2)	0.042 (2)	0.0343 (17)	−0.0149 (18)	−0.0189 (17)	−0.0012 (15)
C36	0.0253 (14)	0.0437 (18)	0.0176 (12)	−0.0011 (13)	−0.0016 (10)	0.0047 (11)
C37	0.060 (3)	0.0362 (19)	0.0387 (18)	−0.0113 (18)	−0.0204 (18)	0.0158 (15)
C38	0.065 (3)	0.0302 (17)	0.0411 (19)	−0.0186 (18)	−0.0265 (19)	0.0135 (14)
C39	0.0246 (13)	0.0246 (13)	0.0197 (11)	0.0020 (11)	0.0040 (10)	0.0019 (10)
C40	0.0298 (16)	0.0277 (15)	0.0335 (15)	−0.0026 (12)	0.0086 (12)	−0.0033 (12)
C41	0.0262 (15)	0.0308 (16)	0.0375 (16)	−0.0006 (12)	0.0104 (12)	−0.0008 (12)
C42	0.0235 (14)	0.0330 (16)	0.0292 (14)	0.0061 (12)	0.0061 (11)	0.0025 (12)
C43	0.0309 (17)	0.0392 (19)	0.0479 (19)	−0.0011 (15)	0.0114 (15)	−0.0167 (15)
C44	0.0260 (15)	0.0368 (17)	0.0435 (18)	−0.0026 (13)	0.0101 (13)	−0.0143 (14)
C45	0.0362 (17)	0.0265 (14)	0.0229 (13)	−0.0057 (12)	0.0022 (12)	−0.0014 (11)
C46	0.0342 (16)	0.0236 (14)	0.0304 (14)	−0.0029 (12)	0.0023 (12)	−0.0040 (11)
C47	0.0362 (18)	0.0328 (17)	0.0399 (17)	−0.0004 (14)	0.0044 (14)	−0.0115 (14)
C48	0.053 (2)	0.052 (2)	0.0261 (15)	0.0001 (19)	0.0047 (15)	−0.0092 (15)
C49	0.0247 (14)	0.0234 (14)	0.0319 (14)	−0.0005 (11)	0.0001 (11)	0.0040 (11)
C50	0.0250 (15)	0.0237 (14)	0.0458 (18)	0.0001 (12)	0.0048 (13)	0.0014 (13)
C51	0.0355 (19)	0.0356 (19)	0.058 (2)	0.0096 (15)	0.0031 (17)	0.0062 (16)
C52B	0.032 (4)	0.040 (4)	0.099 (11)	0.009 (4)	0.002 (6)	0.005 (6)
C52A	0.032 (4)	0.040 (4)	0.099 (11)	0.009 (4)	0.002 (6)	0.005 (6)
C53	0.0236 (14)	0.0286 (15)	0.0470 (18)	−0.0016 (12)	0.0113 (13)	−0.0004 (13)
C54	0.0309 (16)	0.0349 (17)	0.0428 (18)	−0.0074 (14)	0.0138 (14)	−0.0017 (14)
C55	0.0294 (18)	0.050 (2)	0.057 (2)	−0.0026 (16)	0.0138 (16)	0.0101 (18)
C56A	0.049 (4)	0.068 (4)	0.072 (7)	−0.021 (3)	0.024 (5)	0.018 (5)
C56B	0.049 (4)	0.068 (4)	0.072 (7)	−0.021 (3)	0.024 (5)	0.018 (5)
C57	0.0327 (16)	0.0251 (14)	0.0306 (14)	0.0037 (12)	0.0038 (12)	0.0040 (11)
C58	0.0380 (18)	0.0303 (16)	0.0291 (14)	−0.0033 (14)	0.0031 (13)	0.0073 (12)
C59	0.064 (3)	0.0305 (17)	0.0356 (17)	−0.0032 (17)	−0.0052 (17)	0.0076 (14)
C60	0.084 (3)	0.044 (2)	0.0299 (17)	−0.019 (2)	0.0002 (19)	0.0081 (15)
C61	0.0258 (14)	0.0265 (14)	0.0246 (13)	−0.0022 (11)	0.0055 (11)	−0.0001 (11)
C62	0.0349 (17)	0.0308 (16)	0.0300 (14)	−0.0086 (13)	0.0034 (13)	−0.0040 (12)
C63	0.0335 (17)	0.0388 (18)	0.0343 (16)	−0.0124 (15)	−0.0011 (13)	−0.0011 (14)
C64	0.0373 (19)	0.042 (2)	0.0402 (18)	−0.0040 (16)	0.0087 (15)	0.0055 (15)
C65	0.0310 (16)	0.0315 (16)	0.0299 (14)	−0.0006 (13)	0.0100 (12)	−0.0077 (12)
C66	0.047 (2)	0.0336 (17)	0.0233 (13)	−0.0010 (15)	0.0062 (13)	−0.0064 (12)
C67	0.070 (3)	0.059 (3)	0.0304 (17)	0.017 (2)	−0.0035 (18)	−0.0141 (17)
C68	0.055 (3)	0.070 (3)	0.0281 (16)	0.015 (2)	0.0021 (16)	−0.0107 (17)
Cl5	0.0376 (5)	0.0345 (4)	0.0592 (5)	0.0042 (4)	0.0022 (4)	−0.0150 (4)
O1	0.0504 (18)	0.0419 (16)	0.078 (2)	0.0039 (14)	0.0177 (16)	−0.0198 (15)
O2	0.083 (3)	0.047 (2)	0.124 (4)	0.023 (2)	0.033 (3)	0.017 (2)
O3	0.0460 (17)	0.0484 (17)	0.073 (2)	0.0070 (14)	0.0150 (15)	−0.0066 (15)
O4	0.055 (2)	0.151 (5)	0.084 (3)	−0.011 (3)	−0.009 (2)	−0.059 (3)

### Geometric parameters (Å, °)

**Table:**

Co1—N4	1.979 (2)	C42—C43	1.369 (5)
Co1—N2	1.985 (2)	C43—C44	1.395 (5)
Co1—N1	1.989 (2)	C43—H43	0.9500
Co1—N3	1.997 (2)	C44—H44	0.9500
Co1—P2	2.3385 (9)	C45—C46	1.531 (4)
Co1—P1	2.3395 (8)	C45—H45A	0.9900
Cl1—C24	1.753 (3)	C45—H45B	0.9900
Cl2—C30	1.750 (3)	C46—C47	1.536 (5)
Cl3—C36	1.745 (3)	C46—H46A	0.9900
Cl4—C42	1.744 (3)	C46—H46B	0.9900
P1—C49	1.827 (3)	C47—C48	1.521 (5)
P1—C45	1.837 (3)	C47—H47A	0.9900
P1—C53	1.837 (3)	C47—H47B	0.9900
P2—C65	1.836 (3)	C48—H48A	0.9800
P2—C61	1.841 (3)	C48—H48B	0.9800
P2—C57	1.847 (3)	C48—H48C	0.9800
N1—C5	1.376 (4)	C49—C50	1.516 (5)
N1—C2	1.377 (3)	C49—H49A	0.9900
N2—C7	1.378 (4)	C49—H49B	0.9900
N2—C10	1.381 (3)	C50—C51	1.530 (5)
N3—C12	1.373 (3)	C50—H50A	0.9900
N3—C15	1.378 (4)	C50—H50B	0.9900
N4—C17	1.375 (4)	C51—C52A	1.521 (7)
N4—C20	1.386 (3)	C51—C52B	1.537 (10)
C1—C20	1.389 (4)	C51—H51A	0.9900
C1—C2	1.389 (4)	C51—H51B	0.9900
C1—C21	1.502 (4)	C51—H51C	0.9599
C2—C3	1.440 (4)	C51—H51D	0.9601
C3—C4	1.356 (5)	C52B—H52A	0.9800
C3—H3	0.9500	C52B—H52B	0.9800
C4—C5	1.445 (4)	C52B—H52C	0.9800
C4—H4	0.9500	C52A—H52D	0.9800
C5—C6	1.391 (4)	C52A—H52E	0.9800
C6—C7	1.398 (4)	C52A—H52F	0.9800
C6—C27	1.493 (4)	C53—C54	1.510 (5)
C7—C8	1.438 (4)	C53—H53A	0.9900
C8—C9	1.356 (4)	C53—H53B	0.9900
C8—H8	0.9500	C54—C55	1.505 (5)
C9—C10	1.434 (4)	C54—H54A	0.9900
C9—H9	0.9500	C54—H54B	0.9900
C10—C11	1.402 (4)	C55—C56A	1.522 (7)
C11—C12	1.387 (4)	C55—C56B	1.544 (9)
C11—C33	1.503 (4)	C55—H55A	0.9900
C12—C13	1.438 (4)	C55—H55B	0.9900
C13—C14	1.357 (4)	C55—H55C	0.9601
C13—H13	0.9500	C55—H55D	0.9599
C14—C15	1.444 (4)	C56A—H56A	0.9800
C14—H14	0.9500	C56A—H56B	0.9800
C15—C16	1.396 (4)	C56A—H56C	0.9800
C16—C17	1.395 (4)	C56B—H56D	0.9800
C16—C39	1.493 (4)	C56B—H56E	0.9800
C17—C18	1.441 (4)	C56B—H56F	0.9800
C18—C19	1.350 (4)	C57—C58	1.537 (5)
C18—H18	0.9500	C57—H57A	0.9900
C19—C20	1.437 (4)	C57—H57B	0.9900
C19—H19	0.9500	C58—C59	1.529 (5)
C21—C22	1.388 (4)	C58—H58A	0.9900
C21—C26	1.389 (4)	C58—H58B	0.9900
C22—C23	1.391 (4)	C59—C60	1.527 (6)
C22—H22	0.9500	C59—H59A	0.9900
C23—C24	1.366 (5)	C59—H59B	0.9900
C23—H23	0.9500	C60—H60A	0.9800
C24—C25	1.385 (5)	C60—H60B	0.9800
C25—C26	1.393 (4)	C60—H60C	0.9800
C25—H25	0.9500	C61—C62	1.533 (4)
C26—H26	0.9500	C61—H61A	0.9900
C27—C28	1.396 (4)	C61—H61B	0.9900
C27—C32	1.398 (4)	C62—C63	1.546 (5)
C28—C29	1.389 (5)	C62—H62A	0.9900
C28—H28	0.9500	C62—H62B	0.9900
C29—C30	1.390 (5)	C63—C64	1.515 (5)
C29—H29	0.9500	C63—H63A	0.9900
C30—C31	1.372 (5)	C63—H63B	0.9900
C31—C32	1.397 (4)	C64—H64A	0.9800
C31—H31	0.9500	C64—H64B	0.9800
C32—H32	0.9500	C64—H64C	0.9800
C33—C34	1.378 (4)	C65—C66	1.517 (5)
C33—C38	1.378 (4)	C65—H65A	0.9900
C34—C35	1.393 (5)	C65—H65B	0.9900
C34—H34	0.9500	C66—C67	1.508 (5)
C35—C36	1.372 (5)	C66—H66A	0.9900
C35—H35	0.9500	C66—H66B	0.9900
C36—C37	1.368 (5)	C67—C68	1.516 (6)
C37—C38	1.391 (5)	C67—H67A	0.9900
C37—H37	0.9500	C67—H67B	0.9900
C38—H38	0.9500	C68—H68A	0.9800
C39—C44	1.393 (4)	C68—H68B	0.9800
C39—C40	1.395 (4)	C68—H68C	0.9800
C40—C41	1.388 (4)	Cl5—O4	1.410 (4)
C40—H40	0.9500	Cl5—O1	1.432 (3)
C41—C42	1.375 (5)	Cl5—O3	1.440 (3)
C41—H41	0.9500	Cl5—O2	1.453 (4)
			
N4—Co1—N2	178.50 (10)	P1—C45—H45B	107.5
N4—Co1—N1	90.36 (10)	H45A—C45—H45B	107.0
N2—Co1—N1	89.63 (10)	C45—C46—C47	111.3 (3)
N4—Co1—N3	89.92 (9)	C45—C46—H46A	109.4
N2—Co1—N3	90.13 (9)	C47—C46—H46A	109.4
N1—Co1—N3	178.85 (10)	C45—C46—H46B	109.4
N4—Co1—P2	89.41 (7)	C47—C46—H46B	109.4
N2—Co1—P2	89.09 (7)	H46A—C46—H46B	108.0
N1—Co1—P2	91.68 (7)	C48—C47—C46	114.1 (3)
N3—Co1—P2	89.44 (7)	C48—C47—H47A	108.7
N4—Co1—P1	91.40 (7)	C46—C47—H47A	108.7
N2—Co1—P1	90.10 (7)	C48—C47—H47B	108.7
N1—Co1—P1	88.48 (7)	C46—C47—H47B	108.7
N3—Co1—P1	90.39 (7)	H47A—C47—H47B	107.6
P2—Co1—P1	179.18 (3)	C47—C48—H48A	109.5
C49—P1—C45	105.88 (15)	C47—C48—H48B	109.5
C49—P1—C53	104.33 (15)	H48A—C48—H48B	109.5
C45—P1—C53	107.52 (16)	C47—C48—H48C	109.5
C49—P1—Co1	115.86 (11)	H48A—C48—H48C	109.5
C45—P1—Co1	112.48 (10)	H48B—C48—H48C	109.5
C53—P1—Co1	110.16 (11)	C50—C49—P1	116.5 (2)
C65—P2—C61	104.00 (15)	C50—C49—H49A	108.2
C65—P2—C57	100.97 (15)	P1—C49—H49A	108.2
C61—P2—C57	104.32 (15)	C50—C49—H49B	108.2
C65—P2—Co1	118.53 (11)	P1—C49—H49B	108.2
C61—P2—Co1	112.51 (10)	H49A—C49—H49B	107.3
C57—P2—Co1	114.80 (11)	C49—C50—C51	112.3 (3)
C5—N1—C2	105.4 (2)	C49—C50—H50A	109.1
C5—N1—Co1	127.74 (19)	C51—C50—H50A	109.1
C2—N1—Co1	126.86 (19)	C49—C50—H50B	109.1
C7—N2—C10	105.1 (2)	C51—C50—H50B	109.1
C7—N2—Co1	127.51 (18)	H50A—C50—H50B	107.9
C10—N2—Co1	127.38 (19)	C52A—C51—C50	114.6 (11)
C12—N3—C15	105.8 (2)	C50—C51—C52B	106 (2)
C12—N3—Co1	126.70 (19)	C52A—C51—H51A	108.6
C15—N3—Co1	127.35 (18)	C50—C51—H51A	108.6
C17—N4—C20	105.2 (2)	C52B—C51—H51A	102.9
C17—N4—Co1	127.75 (18)	C52A—C51—H51B	108.6
C20—N4—Co1	127.07 (19)	C50—C51—H51B	108.6
C20—C1—C2	123.0 (3)	C52B—C51—H51B	122.8
C20—C1—C21	117.3 (3)	H51A—C51—H51B	107.6
C2—C1—C21	119.7 (2)	C52A—C51—H51C	91.8
N1—C2—C1	126.3 (2)	C50—C51—H51C	111.1
N1—C2—C3	110.4 (3)	C52B—C51—H51C	105.8
C1—C2—C3	123.0 (3)	H51A—C51—H51C	121.4
C4—C3—C2	107.1 (3)	C52A—C51—H51D	117.7
C4—C3—H3	126.5	C50—C51—H51D	110.9
C2—C3—H3	126.5	C52B—C51—H51D	114.2
C3—C4—C5	106.5 (3)	H51B—C51—H51D	94.2
C3—C4—H4	126.7	H51C—C51—H51D	109.1
C5—C4—H4	126.7	C51—C52B—H52A	109.5
N1—C5—C6	126.2 (2)	C51—C52B—H52B	109.5
N1—C5—C4	110.5 (3)	H52A—C52B—H52B	109.5
C6—C5—C4	123.3 (3)	C51—C52B—H52C	109.5
C5—C6—C7	122.4 (3)	H52A—C52B—H52C	109.5
C5—C6—C27	119.1 (2)	C51—C52A—H52D	109.5
C7—C6—C27	118.5 (3)	C51—C52A—H52E	109.5
N2—C7—C6	126.2 (3)	C51—C52A—H52F	109.5
N2—C7—C8	110.6 (2)	C54—C53—P1	119.9 (2)
C6—C7—C8	123.1 (3)	C54—C53—H53A	107.4
C9—C8—C7	106.8 (3)	P1—C53—H53A	107.4
C9—C8—H8	126.6	C54—C53—H53B	107.4
C7—C8—H8	126.6	P1—C53—H53B	107.4
C8—C9—C10	107.0 (2)	H53A—C53—H53B	106.9
C8—C9—H9	126.5	C55—C54—C53	113.0 (3)
C10—C9—H9	126.5	C55—C54—H54A	109.0
N2—C10—C11	125.7 (3)	C53—C54—H54A	109.0
N2—C10—C9	110.5 (2)	C55—C54—H54B	109.0
C11—C10—C9	123.7 (2)	C53—C54—H54B	109.0
C12—C11—C10	122.9 (2)	H54A—C54—H54B	107.8
C12—C11—C33	119.6 (2)	C54—C55—C56A	114.4 (5)
C10—C11—C33	117.5 (3)	C54—C55—C56B	110.5 (9)
N3—C12—C11	126.8 (2)	C54—C55—H55A	108.7
N3—C12—C13	110.3 (3)	C56A—C55—H55A	108.7
C11—C12—C13	122.9 (2)	C56B—C55—H55A	128.8
C14—C13—C12	107.0 (2)	C54—C55—H55B	108.7
C14—C13—H13	126.5	C56A—C55—H55B	108.7
C12—C13—H13	126.5	C56B—C55—H55B	89.7
C13—C14—C15	106.8 (3)	H55A—C55—H55B	107.6
C13—C14—H14	126.6	C54—C55—H55C	109.7
C15—C14—H14	126.6	C56A—C55—H55C	88.6
N3—C15—C16	125.9 (2)	C56B—C55—H55C	110.8
N3—C15—C14	110.0 (2)	H55B—C55—H55C	125.6
C16—C15—C14	124.1 (3)	C54—C55—H55D	109.6
C17—C16—C15	122.9 (3)	C56A—C55—H55D	123.4
C17—C16—C39	116.6 (3)	C56B—C55—H55D	107.9
C15—C16—C39	120.3 (2)	H55A—C55—H55D	88.2
N4—C17—C16	126.2 (3)	H55C—C55—H55D	108.2
N4—C17—C18	110.5 (2)	C55—C56A—H56A	109.5
C16—C17—C18	123.3 (3)	C55—C56A—H56B	109.5
C19—C18—C17	107.0 (3)	C55—C56A—H56C	109.5
C19—C18—H18	126.5	C55—C56B—H56D	109.5
C17—C18—H18	126.5	C55—C56B—H56E	109.5
C18—C19—C20	107.1 (3)	H56D—C56B—H56E	109.5
C18—C19—H19	126.5	C55—C56B—H56F	109.5
C20—C19—H19	126.5	H56D—C56B—H56F	109.5
N4—C20—C1	126.1 (3)	H56E—C56B—H56F	109.5
N4—C20—C19	110.3 (2)	C58—C57—P2	117.2 (2)
C1—C20—C19	123.6 (3)	C58—C57—H57A	108.0
C22—C21—C26	119.2 (3)	P2—C57—H57A	108.0
C22—C21—C1	120.7 (3)	C58—C57—H57B	108.0
C26—C21—C1	120.1 (3)	P2—C57—H57B	108.0
C21—C22—C23	119.9 (3)	H57A—C57—H57B	107.2
C21—C22—H22	120.0	C59—C58—C57	110.8 (3)
C23—C22—H22	120.0	C59—C58—H58A	109.5
C24—C23—C22	119.8 (3)	C57—C58—H58A	109.5
C24—C23—H23	120.1	C59—C58—H58B	109.5
C22—C23—H23	120.1	C57—C58—H58B	109.5
C23—C24—C25	121.8 (3)	H58A—C58—H58B	108.1
C23—C24—Cl1	119.3 (3)	C60—C59—C58	111.0 (4)
C25—C24—Cl1	118.9 (3)	C60—C59—H59A	109.4
C24—C25—C26	118.0 (3)	C58—C59—H59A	109.4
C24—C25—H25	121.0	C60—C59—H59B	109.4
C26—C25—H25	121.0	C58—C59—H59B	109.4
C21—C26—C25	121.2 (3)	H59A—C59—H59B	108.0
C21—C26—H26	119.4	C59—C60—H60A	109.5
C25—C26—H26	119.4	C59—C60—H60B	109.5
C28—C27—C32	118.9 (3)	H60A—C60—H60B	109.5
C28—C27—C6	120.6 (3)	C59—C60—H60C	109.5
C32—C27—C6	120.5 (3)	H60A—C60—H60C	109.5
C29—C28—C27	121.2 (3)	H60B—C60—H60C	109.5
C29—C28—H28	119.4	C62—C61—P2	116.5 (2)
C27—C28—H28	119.4	C62—C61—H61A	108.2
C28—C29—C30	118.4 (3)	P2—C61—H61A	108.2
C28—C29—H29	120.8	C62—C61—H61B	108.2
C30—C29—H29	120.8	P2—C61—H61B	108.2
C31—C30—C29	122.0 (3)	H61A—C61—H61B	107.3
C31—C30—Cl2	119.7 (3)	C61—C62—C63	111.4 (3)
C29—C30—Cl2	118.3 (3)	C61—C62—H62A	109.4
C30—C31—C32	119.1 (3)	C63—C62—H62A	109.4
C30—C31—H31	120.4	C61—C62—H62B	109.4
C32—C31—H31	120.4	C63—C62—H62B	109.4
C31—C32—C27	120.4 (3)	H62A—C62—H62B	108.0
C31—C32—H32	119.8	C64—C63—C62	112.7 (3)
C27—C32—H32	119.8	C64—C63—H63A	109.0
C34—C33—C38	118.2 (3)	C62—C63—H63A	109.0
C34—C33—C11	119.6 (3)	C64—C63—H63B	109.0
C38—C33—C11	122.2 (3)	C62—C63—H63B	109.0
C33—C34—C35	121.4 (3)	H63A—C63—H63B	107.8
C33—C34—H34	119.3	C63—C64—H64A	109.5
C35—C34—H34	119.3	C63—C64—H64B	109.5
C36—C35—C34	118.9 (3)	H64A—C64—H64B	109.5
C36—C35—H35	120.5	C63—C64—H64C	109.5
C34—C35—H35	120.5	H64A—C64—H64C	109.5
C37—C36—C35	121.0 (3)	H64B—C64—H64C	109.5
C37—C36—Cl3	119.2 (3)	C66—C65—P2	116.8 (2)
C35—C36—Cl3	119.8 (3)	C66—C65—H65A	108.1
C36—C37—C38	119.2 (3)	P2—C65—H65A	108.1
C36—C37—H37	120.4	C66—C65—H65B	108.1
C38—C37—H37	120.4	P2—C65—H65B	108.1
C33—C38—C37	121.2 (3)	H65A—C65—H65B	107.3
C33—C38—H38	119.4	C67—C66—C65	113.2 (3)
C37—C38—H38	119.4	C67—C66—H66A	108.9
C44—C39—C40	117.8 (3)	C65—C66—H66A	108.9
C44—C39—C16	119.5 (3)	C67—C66—H66B	108.9
C40—C39—C16	122.4 (3)	C65—C66—H66B	108.9
C41—C40—C39	121.2 (3)	H66A—C66—H66B	107.7
C41—C40—H40	119.4	C66—C67—C68	112.1 (4)
C39—C40—H40	119.4	C66—C67—H67A	109.2
C42—C41—C40	119.2 (3)	C68—C67—H67A	109.2
C42—C41—H41	120.4	C66—C67—H67B	109.2
C40—C41—H41	120.4	C68—C67—H67B	109.2
C43—C42—C41	121.4 (3)	H67A—C67—H67B	107.9
C43—C42—Cl4	119.2 (3)	C67—C68—H68A	109.5
C41—C42—Cl4	119.4 (3)	C67—C68—H68B	109.5
C42—C43—C44	119.1 (3)	H68A—C68—H68B	109.5
C42—C43—H43	120.4	C67—C68—H68C	109.5
C44—C43—H43	120.4	H68A—C68—H68C	109.5
C39—C44—C43	121.2 (3)	H68B—C68—H68C	109.5
C39—C44—H44	119.4	O4—Cl5—O1	110.1 (2)
C43—C44—H44	119.4	O4—Cl5—O3	110.1 (3)
C46—C45—P1	119.2 (2)	O1—Cl5—O3	110.2 (2)
C46—C45—H45A	107.5	O4—Cl5—O2	111.1 (3)
P1—C45—H45A	107.5	O1—Cl5—O2	106.7 (2)
C46—C45—H45B	107.5	O3—Cl5—O2	108.6 (2)
			
N4—Co1—P1—C49	−29.06 (14)	C13—C14—C15—C16	−177.3 (3)
N2—Co1—P1—C49	150.88 (14)	N3—C15—C16—C17	0.2 (5)
N1—Co1—P1—C49	61.26 (14)	C14—C15—C16—C17	178.7 (3)
N3—Co1—P1—C49	−118.99 (14)	N3—C15—C16—C39	−175.2 (3)
N4—Co1—P1—C45	92.92 (14)	C14—C15—C16—C39	3.2 (5)
N2—Co1—P1—C45	−87.14 (14)	C20—N4—C17—C16	−177.3 (3)
N1—Co1—P1—C45	−176.77 (14)	Co1—N4—C17—C16	3.2 (4)
N3—Co1—P1—C45	2.99 (14)	C20—N4—C17—C18	−0.2 (3)
N4—Co1—P1—C53	−147.15 (14)	Co1—N4—C17—C18	−179.7 (2)
N2—Co1—P1—C53	32.79 (14)	C15—C16—C17—N4	−2.4 (5)
N1—Co1—P1—C53	−56.83 (14)	C39—C16—C17—N4	173.2 (3)
N3—Co1—P1—C53	122.92 (14)	C15—C16—C17—C18	−179.2 (3)
N4—Co1—P2—C65	104.68 (14)	C39—C16—C17—C18	−3.5 (4)
N2—Co1—P2—C65	−75.26 (14)	N4—C17—C18—C19	0.2 (3)
N1—Co1—P2—C65	14.34 (15)	C16—C17—C18—C19	177.4 (3)
N3—Co1—P2—C65	−165.40 (15)	C17—C18—C19—C20	−0.1 (3)
N4—Co1—P2—C61	−16.89 (12)	C17—N4—C20—C1	177.6 (3)
N2—Co1—P2—C61	163.18 (12)	Co1—N4—C20—C1	−2.9 (4)
N1—Co1—P2—C61	−107.22 (12)	C17—N4—C20—C19	0.2 (3)
N3—Co1—P2—C61	73.04 (12)	Co1—N4—C20—C19	179.7 (2)
N4—Co1—P2—C57	−135.99 (13)	C2—C1—C20—N4	1.4 (5)
N2—Co1—P2—C57	44.07 (13)	C21—C1—C20—N4	−179.6 (3)
N1—Co1—P2—C57	133.67 (14)	C2—C1—C20—C19	178.5 (3)
N3—Co1—P2—C57	−46.07 (14)	C21—C1—C20—C19	−2.4 (4)
N4—Co1—N1—C5	−175.6 (3)	C18—C19—C20—N4	−0.1 (3)
N2—Co1—N1—C5	2.9 (3)	C18—C19—C20—C1	−177.6 (3)
P2—Co1—N1—C5	−86.2 (2)	C20—C1—C21—C22	100.4 (4)
P1—Co1—N1—C5	93.0 (2)	C2—C1—C21—C22	−80.6 (4)
N4—Co1—N1—C2	4.6 (2)	C20—C1—C21—C26	−77.6 (4)
N2—Co1—N1—C2	−176.9 (2)	C2—C1—C21—C26	101.5 (4)
P2—Co1—N1—C2	94.0 (2)	C26—C21—C22—C23	2.1 (5)
P1—Co1—N1—C2	−86.8 (2)	C1—C21—C22—C23	−175.8 (3)
N1—Co1—N2—C7	−5.7 (2)	C21—C22—C23—C24	−0.7 (6)
N3—Co1—N2—C7	175.5 (2)	C22—C23—C24—C25	−1.2 (6)
P2—Co1—N2—C7	86.0 (2)	C22—C23—C24—Cl1	178.6 (3)
P1—Co1—N2—C7	−94.1 (2)	C23—C24—C25—C26	1.6 (6)
N1—Co1—N2—C10	172.9 (2)	Cl1—C24—C25—C26	−178.2 (3)
N3—Co1—N2—C10	−6.0 (2)	C22—C21—C26—C25	−1.7 (5)
P2—Co1—N2—C10	−95.5 (2)	C1—C21—C26—C25	176.3 (3)
P1—Co1—N2—C10	84.4 (2)	C24—C25—C26—C21	−0.1 (5)
N4—Co1—N3—C12	−175.6 (2)	C5—C6—C27—C28	93.9 (4)
N2—Co1—N3—C12	5.9 (2)	C7—C6—C27—C28	−83.4 (4)
P2—Co1—N3—C12	95.0 (2)	C5—C6—C27—C32	−86.2 (4)
P1—Co1—N3—C12	−84.2 (2)	C7—C6—C27—C32	96.5 (3)
N4—Co1—N3—C15	−0.3 (2)	C32—C27—C28—C29	−0.8 (5)
N2—Co1—N3—C15	−178.8 (2)	C6—C27—C28—C29	179.1 (3)
P2—Co1—N3—C15	−89.7 (2)	C27—C28—C29—C30	0.2 (5)
P1—Co1—N3—C15	91.1 (2)	C28—C29—C30—C31	0.4 (5)
N1—Co1—N4—C17	179.5 (2)	C28—C29—C30—Cl2	−178.4 (3)
N3—Co1—N4—C17	−1.6 (2)	C29—C30—C31—C32	−0.4 (5)
P2—Co1—N4—C17	87.8 (2)	Cl2—C30—C31—C32	178.4 (2)
P1—Co1—N4—C17	−92.0 (2)	C30—C31—C32—C27	−0.2 (5)
N1—Co1—N4—C20	0.1 (2)	C28—C27—C32—C31	0.8 (4)
N3—Co1—N4—C20	179.0 (2)	C6—C27—C32—C31	−179.1 (3)
P2—Co1—N4—C20	−91.5 (2)	C12—C11—C33—C34	−94.3 (4)
P1—Co1—N4—C20	88.6 (2)	C10—C11—C33—C34	84.1 (4)
C5—N1—C2—C1	172.8 (3)	C12—C11—C33—C38	85.5 (4)
Co1—N1—C2—C1	−7.3 (4)	C10—C11—C33—C38	−96.1 (4)
C5—N1—C2—C3	−1.5 (3)	C38—C33—C34—C35	2.4 (6)
Co1—N1—C2—C3	178.4 (2)	C11—C33—C34—C35	−177.8 (4)
C20—C1—C2—N1	4.0 (5)	C33—C34—C35—C36	−0.4 (7)
C21—C1—C2—N1	−175.1 (3)	C34—C35—C36—C37	−1.8 (7)
C20—C1—C2—C3	177.6 (3)	C34—C35—C36—Cl3	179.3 (3)
C21—C1—C2—C3	−1.4 (5)	C35—C36—C37—C38	1.9 (7)
N1—C2—C3—C4	1.5 (4)	Cl3—C36—C37—C38	−179.2 (4)
C1—C2—C3—C4	−173.1 (3)	C34—C33—C38—C37	−2.3 (7)
C2—C3—C4—C5	−0.7 (4)	C11—C33—C38—C37	178.0 (4)
C2—N1—C5—C6	−178.5 (3)	C36—C37—C38—C33	0.2 (7)
Co1—N1—C5—C6	1.6 (4)	C17—C16—C39—C44	−100.2 (4)
C2—N1—C5—C4	1.0 (3)	C15—C16—C39—C44	75.5 (4)
Co1—N1—C5—C4	−178.8 (2)	C17—C16—C39—C40	74.3 (4)
C3—C4—C5—N1	−0.2 (4)	C15—C16—C39—C40	−110.0 (3)
C3—C4—C5—C6	179.4 (3)	C44—C39—C40—C41	1.7 (5)
N1—C5—C6—C7	−5.1 (5)	C16—C39—C40—C41	−172.9 (3)
C4—C5—C6—C7	175.4 (3)	C39—C40—C41—C42	−0.6 (5)
N1—C5—C6—C27	177.8 (3)	C40—C41—C42—C43	−1.0 (5)
C4—C5—C6—C27	−1.7 (4)	C40—C41—C42—Cl4	178.5 (3)
C10—N2—C7—C6	−174.5 (3)	C41—C42—C43—C44	1.4 (6)
Co1—N2—C7—C6	4.3 (4)	Cl4—C42—C43—C44	−178.1 (3)
C10—N2—C7—C8	1.6 (3)	C40—C39—C44—C43	−1.3 (5)
Co1—N2—C7—C8	−179.6 (2)	C16—C39—C44—C43	173.4 (3)
C5—C6—C7—N2	2.0 (5)	C42—C43—C44—C39	−0.2 (6)
C27—C6—C7—N2	179.1 (3)	C49—P1—C45—C46	−49.8 (3)
C5—C6—C7—C8	−173.6 (3)	C53—P1—C45—C46	61.3 (3)
C27—C6—C7—C8	3.5 (4)	Co1—P1—C45—C46	−177.2 (2)
N2—C7—C8—C9	−1.9 (3)	P1—C45—C46—C47	162.2 (3)
C6—C7—C8—C9	174.3 (3)	C45—C46—C47—C48	60.9 (4)
C7—C8—C9—C10	1.3 (3)	C45—P1—C49—C50	−44.9 (3)
C7—N2—C10—C11	−178.2 (3)	C53—P1—C49—C50	−158.2 (2)
Co1—N2—C10—C11	3.0 (4)	Co1—P1—C49—C50	80.6 (2)
C7—N2—C10—C9	−0.8 (3)	P1—C49—C50—C51	−173.1 (3)
Co1—N2—C10—C9	−179.6 (2)	C49—C50—C51—C52A	172.0 (13)
C8—C9—C10—N2	−0.4 (3)	C49—C50—C51—C52B	−176 (2)
C8—C9—C10—C11	177.1 (3)	C49—P1—C53—C54	47.7 (3)
N2—C10—C11—C12	2.8 (5)	C45—P1—C53—C54	−64.5 (3)
C9—C10—C11—C12	−174.3 (3)	Co1—P1—C53—C54	172.6 (3)
N2—C10—C11—C33	−175.5 (3)	P1—C53—C54—C55	−173.7 (3)
C9—C10—C11—C33	7.4 (4)	C53—C54—C55—C56A	169.8 (7)
C15—N3—C12—C11	−178.9 (3)	C53—C54—C55—C56B	−165.5 (11)
Co1—N3—C12—C11	−2.8 (4)	C65—P2—C57—C58	−147.6 (3)
C15—N3—C12—C13	1.2 (3)	C61—P2—C57—C58	−39.9 (3)
Co1—N3—C12—C13	177.4 (2)	Co1—P2—C57—C58	83.7 (3)
C10—C11—C12—N3	−2.9 (5)	P2—C57—C58—C59	178.0 (2)
C33—C11—C12—N3	175.4 (3)	C57—C58—C59—C60	174.7 (3)
C10—C11—C12—C13	177.0 (3)	C65—P2—C61—C62	47.5 (3)
C33—C11—C12—C13	−4.8 (4)	C57—P2—C61—C62	−58.0 (3)
N3—C12—C13—C14	−0.4 (4)	Co1—P2—C61—C62	176.98 (19)
C11—C12—C13—C14	179.7 (3)	P2—C61—C62—C63	166.8 (2)
C12—C13—C14—C15	−0.6 (4)	C61—C62—C63—C64	−70.9 (4)
C12—N3—C15—C16	177.0 (3)	C61—P2—C65—C66	56.3 (3)
Co1—N3—C15—C16	0.9 (4)	C57—P2—C65—C66	164.2 (3)
C12—N3—C15—C14	−1.6 (3)	Co1—P2—C65—C66	−69.5 (3)
Co1—N3—C15—C14	−177.7 (2)	P2—C65—C66—C67	−156.2 (3)
C13—C14—C15—N3	1.4 (4)	C65—C66—C67—C68	−171.5 (4)

### Hydrogen-bond geometry (Å, °)

**Table:**

*D*—H···*A*	*D*—H	H···*A*	*D*···*A*	*D*—H···*A*
C22—H22···O3^i^	0.95	2.48	3.403 (5)	165
C31—H31···O3	0.95	2.55	3.277 (5)	134
C32—H32···O1	0.95	2.49	3.407 (5)	163
C34—H34···Cl4^ii^	0.95	2.80	3.643 (4)	148
C50—H50B···N4	0.99	2.59	3.359 (4)	134
C67—H67B···O1	0.99	2.52	3.376 (6)	145
C66—H66B···Cg1	0.99	2.43	3.335 (4)	151
C53—H53A···Cg2	0.99	2.89	3.574 (4)	127
C58—H58A···Cg3	0.99	2.48	3.431 (4)	161
C50—H50B···Cg4	0.99	2.47	3.353 (4)	149
C61—H61B···Cg4	0.99	2.77	3.428 (3)	125

Symmetry codes: (i) −*x*+1, *y*+1/2, −*z*+3/2; (ii) *x*+1, *y*, *z*.
